# Prognostic Value of Left Ventricular End-Diastolic Pressure in Patients With Non-ST-Segment Elevation Myocardial Infarction

**DOI:** 10.14740/cr406w

**Published:** 2015-10-25

**Authors:** Akihiro Kobayashi, Naoki Misumida, John T. Fox, Yumiko Kanei

**Affiliations:** a Department of Internal Medicine, Mount Sinai Beth Israel, New York, USA; b Department of Cardiology, Mount Sinai Beth Israel, New York, USA

**Keywords:** Left ventricular end-diastolic pressure, Non-ST-segment elevation myocardial infarction, Acute coronary syndrome, Heart failure, Mortality

## Abstract

**Background:**

Elevated left ventricular end-diastolic pressure (LVEDP) has been reported to predict an increased mortality in patients with ST-segment elevation myocardial infarction. However, its prognostic value in patients with non-ST-segment elevation myocardial infarction (NSTEMI) remains unclear.

**Methods:**

We performed a retrospective analysis of NSTEMI patients who underwent coronary angiography between January 2013 and June 2014. We excluded patients who did not undergo LVEDP measurements. Baseline and angiographic characteristics, in-hospital heart failure as well as in-hospital mortality were recorded.

**Results:**

After exclusion, 367 patients were included in the final analysis. The median (interquartile range) LVEDP was 19 mm Hg (14 - 24 mm Hg). By receiver operating characteristic curve analysis, the optimal cutoff value for predicting in-hospital mortality was 22 mm Hg (area under the curve 0.80, sensitivity 80%, and specificity 71%). Of 367 patients, 109 patients (29.7%) had LVEDP > 22 mm Hg. Patients with LVEDP > 22 mm Hg had a greater number of comorbidities. There was no statistically significant difference in the rate of multi-vessel disease. Patients with LVEDP > 22 mm Hg had a significantly higher rate of in-hospital heart failure (22.0% vs. 13.2%, P = 0.03) and in-hospital mortality (3.7% vs. 0.4%, P = 0.03) than those with LVEDP ≤ 22 mm Hg.

**Conclusion:**

Elevated LVEDP was significantly associated with a higher in-hospital mortality in patients with NSTEMI.

## Introduction

Acute myocardial infarction affects both systolic and diastolic function of the left ventricle [1]. Left ventricular ejection fraction (LVEF), which reflects left ventricular systolic function, has been shown to predict unfavorable outcomes in patients with acute myocardial infarction [2, 3].

Left ventricular end-diastolic pressure (LVEDP) elevates in the setting of acute myocardial infarction, as consequent myocardial edema due to ischemia leads to stiffening of the myocardial wall and decreased left ventricular global compliance [4]. Elevated LVEDP has been reported to predict both in-hospital and long-term mortalities in patients with ST-segment elevation myocardial infarction (STEMI) [5, 6]. A previous study on an acute coronary syndrome population showed that although elevated LVEDP was an independent predictor for long-term mortality, its impact on in-hospital mortality did not reach a statistical significance [7]. The prognostic value of LVEDP has not been previously addressed in a specific non-ST-segment elevation myocardial infarction (NSTEMI) population.

We hypothesized that elevated LVEDP predicts in-hospital mortality in an NSTEMI population, which has been shown to hold a higher mortality compared to patients with unstable angina [8]. Thus, the aim of this study was to evaluate the prognostic value of LVEDP in patients with NSTEMI.

## Methods

A retrospective analysis was performed on NSTEMI patients who underwent coronary angiography between January 2013 and June 2014 at our institution. Myocardial infarction was diagnosed in accordance with the European Society of Cardiology and American College of Cardiology criteria [9]. Inclusion criteria were: 1) troponin I level greater than the 99th percentile reference value before cardiac catheterization; 2) chest pain (or anginal equivalent) or ischemic change on electrocardiogram including horizontal or down-sloping ST-segment depression (≥ 0.05 mV) or T-wave inversion (≥ 0.1 mV) in two or more contiguous leads; and 3) the absence of ST-segment elevation and new left bundle branch block on electrocardiogram. Exclusion criteria were: 1) cardiac catheterization more than 5 days after presentation; 2) other identifiable causes of troponin elevation including Takotsubo cardiomyopathy, myocarditis, and pulmonary embolism; and 3) insufficient data for analysis. The present study complied with the Declaration of Helsinki and was approved by the institutional review board of our hospital.

### Demographic, hemodynamic, and laboratory data

Patients’ demographic data, risk factors and hemodynamic parameters such as blood pressure, heart rate, and Killip classification were obtained. Laboratory data on admission including white blood cell count, hemoglobin level, creatinine, and cardiac troponin I (cTnI) were recorded. cTnI level was measured using the second-generation VITROS^®^ troponin I assay (Ortho-Clinical Diagnostics Inc., NJ, USA). The upper limit of normal for cTnI was 0.034 µg/L, which represented the 99th percentile reference value. The highest level was designated as peak cTnI. LVEF was evaluated during hospital stay either with transthoracic echocardiography or with ventriculography.

### Coronary angiography and LVEDP measurement

All patients underwent cardiac catheterization within 5 days of presentation. An independent cardiologist blinded to the clinical data interpreted all coronary angiography findings visually, and the assessment was compared to the primary assessment by the treating cardiologist. In the event of a discrepancy between the assessments, a third investigator made the final interpretation. Obstructive CAD was defined as stenosis greater than or equal to 50% in the left main coronary artery and 70% in any other epicardial coronary arteries. Revascularization procedures including percutaneous coronary intervention (PCI) and coronary artery bypass grafting (CABG) were performed at the discretion of the treating physician. Coronary blood flow was graded according to thrombolysis in myocardial infarction (TIMI) criteria [10]. LVEDP was measured during index cardiac catheterization procedure using inherent software on our angiography system (Sensis Hemodynamic Recording System, VC12B software, Siemens Medical Systems, PA, USA).

### End points

The primary end point for this study was in-hospital all-cause mortality. The secondary end point was in-hospital heart failure defined as the presence of either a heart failure symptom (shortness of breath or orthopnea) or a sign of heart failure (edema or rales on the physical exam) in addition to pulmonary vascular congestion on chest radiography.

### Statistic analyses

Data were expressed as either a number (percentage) or median (interquartile range). Continuous variables were compared using the Wilcoxon rank sum test and dichotomous variables were compared using the Chi-squared test or Fisher’s exact test, as appropriate. Receiver operating characteristic curve analysis was constructed to determine the optimal LVEDP cutoff value for predicting in-hospital mortality and patients were divided into two corresponding groups. In addition, linear correlation between LVEDP and LVEF was evaluated using the Spearman correlation coefficient. Two-sided P values < 0.05 were considered statistically significant. All statistical analyses were performed using R software (version 3.0.1).

## Results

According to the inclusion and exclusion criteria, 481 NSTEMI patients were identified, 114 of which without LVEDP data were excluded. Thus, a total of 367 NSTEMI patients were included in the final analysis. No statistically significant difference was observed either in baseline characteristics or in-hospital mortality between patients with and without LVEDP measurements.

The median (interquartile range) LVEDP was 19 mm Hg (14 - 24 mm Hg). By receiver operating characteristics curve analysis, the optimal cutoff value of LVEDP for predicting in-hospital mortality was 22 mm Hg (area under the curve 0.80, sensitivity 80%, and specificity 71%). Among 367 patients, 109 patients (29.7%) had LVEDP > 22 mg Hg and 258 patients (70.3%) had LVEDP ≤ 22 mm Hg.

Demographic, hemodynamic and laboratory characteristics are summarized and presented in Table 1. Compared to patients with LVEDP ≤ 22 mm Hg, patients with LVEDP > 22 mm Hg were more likely to have a high body mass index, hypertension, diabetes mellitus, chronic kidney disease, and previous revascularization. Patients with LVEDP > 22 mm Hg had a higher, albeit statistically insignificant, rate of history of heart failure and previous myocardial infarction compared to patients with LVEDP ≤ 22 mm Hg. Patients with LVEDP > 22 mm Hg had a higher peak troponin I value and lower LVEF than those with LVEDP ≤ 22 mm Hg. Spearman correlation analysis demonstrated a weak negative correlation between LVEDP and LVEF (r = -0.16, P = 0.002).

**Table 1:** Demographic, Hemodynamic and Laboratory Characteristics

	LVEDP > 22 mm Hg (n = 109)	LVEDP ≤ 22 mm Hg (n = 258)	P value
Demographics			
Age (years)	64 (54 - 74)	66 (57 - 75)	0.35
Male	68 (62)	162 (63)	0.94
Body mass index (kg/m^2^)	29.5 (25.6 - 33.1)	26.5 (23.3 - 29.7)	< 0.001
Hypertension	87 (80)	178 (69)	0.03
Diabetes mellitus	55 (50)	85 (33)	0.001
Hyperlipidemia	64 (59)	138 (53)	0.35
Chronic kidney disease*	45 (41)	68 (26)	0.005
Family history of coronary artery disease	28 (26)	51 (20)	0.21
Current smoker	19 (17)	65 (25)	0.11
History of heart failure	15 (14)	19 (7)	0.053
Previous PCI	37 (34)	59 (23)	0.027
Previous CABG	20 (18)	17 (7)	< 0.001
Previous myocardial infarction	22 (20)	32 (12)	0.055
TIMI risk score			0.23
Low risk (0 - 2)	17 (16)	57 (22)	
Intermediate risk (3 - 4)	59 (54)	140 (54)	
High risk (5 - 7)	33 (30)	61 (24)	
Hemodynamic and laboratory data			
Systolic blood pressure (mm Hg)	144 (127 - 160)	144 (126 - 159)	0.63
Diastolic blood pressure (mm Hg)	80 (71 - 91)	81 (73 - 93)	0.71
Heart rate (beat/min)	85 (72 - 97)	80 (70 - 92)	0.08
Hemoglobin (g/L)	12.8 (11.3 - 14.1)	13.3 (12.2 - 14.5)	0.01
White blood cell count (10^9^/L)	8.7 (7.0 - 10.9)	8.5 (6.7 - 10.7)	0.76
eGFR (mL/min/1.73 m^2^)	65 (45 - 81)	78 (58 - 93)	0.001
Peak troponin I (μg/L)	1.64 (0.15 - 7.54)	0.55 (0.10 - 5.39)	0.049
Killip class on admission II-IV	22 (20)	30 (12)	0.032
Left ventricular ejection fraction (%)	52 (33 - 60)	60 (50 - 65)	< 0.001

Data are expressed as a number (percent) or median (interquartile range). LVEDP: left ventricular end-diastolic pressure; PCI: percutaneous coronary intervention; CABG: coronary artery bypass grafting; TIMI: thrombolysis in myocardial infarction; eGFR: estimated glomerular filtration rate. *Chronic kidney disease was defined as eGFR < 60 mL/min/1.73 m^2^.

Angiographic characteristics, in-hospital revascularization procedures, and in-hospital outcomes are summarized and presented in Table 2. There was no significant difference in number of diseased vessels or pre-procedural coronary blood flow of the infarct-related artery between the two groups. No statistically significant difference was found in the rate of in-hospital PCI or CABG between patients with LVEDP > 22 mm Hg and those with LVEDP ≤ 22 mm Hg. Patients with LVEDP > 22 mm Hg had a significantly higher rate of in-hospital heart failure and in-hospital mortality than those with LVEDP ≤ 22 mm Hg.

**Table 2:** Angiographic Characteristics, In-Hospital Revascularization Procedures, and In-Hospital Outcomes

	LVEDP > 22 mm Hg (n = 109)	LVEDP ≤ 22 mm Hg (n = 258)	P value
Angiographic findings			
Interval to angiography from hospital admission (days)	0.95 (0.57 - 1.77)	1.21 (0.67 - 2.20)	0.017
LAD coronary artery culprit lesion	28 (26)	60 (23)	0.62
Multi-vessel disease	59 (54)	138 (53)	0.91
Collateral flow to infarcted related artery	37 (34)	65 (25)	0.087
Number of diseased vessels			
0	22 (20)	57 (22)	0.68
1	28 (26)	63 (24)	0.8
2	31 (28)	72 (28)	0.92
3	28 (26)	66 (26)	0.98
Pre-procedural TIMI flow grade			0.66
0/1	29 (27)	63 (24)	
2/3	80 (73)	195 (76)	
In-hospital PCI	49 (45)	136 (53)	0.17
In-hospital CABG	8 (7)	28 (11)	0.3
In-hospital outcomes			
All-cause mortality	4 (4)	1 (0.4)	0.029
Heart failure	24 (22)	34 (13)	0.034

Data are expressed as a number (percent) or median (interquartile range). LVEDP: left ventricular end-diastolic pressure; LAD: left anterior descending; TIMI: thrombolysis in myocardial infarction; PCI: percutaneous coronary intervention; CABG: coronary artery bypass grafting.

## Discussion

Our study has shown that elevated LVEDP defined as LVEDP > 22 mm Hg is significantly associated with a higher in-hospital heart failure and in-hospital mortality in patients with NSTEMI. Elevated LVEDP has been shown to predict both in-hospital and long-term mortalities in patients with STEMI [5, 6]. Teixeira et al evaluated the prognostic value of LVEDP in an acute coronary syndrome population that consisted of STEMI (43%), NSTEMI (35.8%), and unstable angina (18.8%) patients. They reported that elevated LVEDP was an independent predictor of long-term mortality. However, its impact on in-hospital mortality did not reach a statistical significance.

In the present study, we specifically included patients with NSTEMI, who have a higher in-hospital mortality than those with unstable angina [8]. In our study, LVEDP was measured in 76.3% of all eligible patients in contrast to 59.1% in Teixeira’s study. The higher rate of LVEDP measurements and inclusion of specific NSTEMI patients would have yielded a more specific prognostic value of LVEDP in the NSTEMI population.

The higher in-hospital mortality associated with elevated LVEDP could be attributed to the higher rate of concomitant in-hospital heart failure, which has been shown to be associated with a four-fold increase in in-hospital mortality in patients with acute coronary syndrome [11]. Elevated LVEDP has been reported to affect coronary perfusion in myocardium of the infarcted area in patients with acute myocardial infarction [12]. In addition, elevated LVEDP represented a high-risk population in our study as evidenced by increased incidence of hypertension, diabetes mellitus, and chronic kidney disease. These high-risk characteristics can also explain the higher in-hospital mortality in patients with elevated LVEDP. Our present study suggests that LVEDP is a useful hemodynamic parameter for stratifying high-risk patients in the NSTEMI population.

This study has several limitations, including a retrospective design, a relatively small number of patients, and the lack of data on long-term clinical events. In addition, the low in-hospital mortality in our cohort did not allow us to evaluate the independent prognostic value of LVEDP.

In conclusion, the present study shows that elevated LVEDP is significantly associated with higher in-hospital mortality in patients with NSTEMI, suggesting that LVEDP is a useful hemodynamic parameter for stratifying high-risk patients in the NSTEMI population.
